# Hypoaminoacidemia and Pyroglutamic Aciduria: Potential Biomarkers in Malnutrition‐Related Hyperammonemia

**DOI:** 10.1002/jmd2.70058

**Published:** 2026-01-18

**Authors:** M. M. Crenshaw, O. M. D'Annibale, V. Martucci, S. Gracie, A. Kochhar, J. Stansauk, A. Larson, P. Baker, C. Peck, T. Wood, A. El‐Gharbawy, S. McCandless, M. K. LoPiccolo

**Affiliations:** ^1^ Department of Pediatrics, Section of Clinical Genetics and Metabolism University of Colorado School of Medicine Aurora Colorado USA; ^2^ Division of Genetics & Metabolism, Department of Pediatrics University of North Carolina at Chapel Hill Chapel Hill North Carolina USA; ^3^ Biochemical Genetics Laboratory Children's Hospital Colorado Anschutz Medical Campus Aurora Colorado USA; ^4^ Division of Human Genetics, Department of Pediatrics University of Cincinnati College of Medicine Cincinnati Ohio USA; ^5^ Department of Pediatrics, Section of Cardiology University of Colorado School of Medicine Aurora Colorado USA; ^6^ Division of Medical Genetics, Department of Pediatrics Duke University Medical Center Durham North Carolina USA; ^7^ Department of Genetics and Genomics Sciences Icahn School of Medicine at Mount Sinai New York New York USA

**Keywords:** hyperammonemia, hypoaminoacidemia, malnutrition, pyroglutamic aciduria

## Abstract

Hyperammonemia is a medical emergency, and the cause must be identified quickly in order to treat appropriately. Malnutrition is a known risk factor for hyperammonemia; however, there are limited reliable lab indicators used to identify malnutrition. Early identification of the etiology of hyperammonemia is crucial to optimizing care, specifically reintroduction of appropriate amounts of protein into the diet. Herein, we discuss three patients with complex medical histories and clinical signs of malnutrition who presented with hyperammonemia. In all three patients, both hypoaminoacidemia and pyroglutamic aciduria were observed. Specifically, all patients had low tyrosine, tryptophan, methionine, and branched‐chain amino acids. Recognizing this biochemical pattern could result in more rapid initiation of supplementing protein, a primary tenet of treatment in malnutrition‐related hyperammonemia. We highlight the unique features of malnutrition‐related hyperammonemia, propose mechanisms to explain the pattern, and suggest a framework for managing these cases.

## Synopsis

We present three patients who underwent a metabolic work‐up for hyperammonemia, later found to have malnutrition‐related hyperammonemia and a biochemical pattern of hypoaminoacidemia and pyroglutamic aciduria suggesting that this pattern could be used to indicate the diagnosis earlier and therefore allow for more rapid initiation of protein supplementation.

## 1 Introduction

Hyperammonemia is a medical emergency that can present at any age with encephalopathy and the risk of progression to permanent neurologic damage and death [1]. A variety of underlying etiologies for hyperammonemia exist, each with unique treatments; thus, early identification of the cause of hyperammonemia is critical.

Hepatic causes of hyperammonemia include liver failure, cirrhosis, and urea cycle disorders [1]. The etiology of hyperammonemia can be broadly separated into two distinct mechanisms: increased ammonia production and decreased ammonia clearance [2]. Increased ammonia production may be due to high dietary protein intake, infections, namely intestinal bacterial overgrowth and urease‐producing bacterial infections, increased cell turnover from oncologic disease, severe muscle breakdown, as in the setting of status epilepticus, or treatment with specific chemotherapeutics [1, 2, 3, 4, 5, 6, 7]. Decreased ammonia clearance can result from portosystemic shunting, renal dysfunction, or primary and secondary urea cycle dysfunction [1, 2]. Moreover, medications, including valproic acid, salicylates, and gabapentin, have been found to reduce ammonia clearance [1, 2]. Other medical interventions associated with an increased risk of hyperammonemia include bone marrow or solid organ transplants and bariatric surgery [5, 6, 8, 9].

In the body, ammonium ion (NH_4_^+^) and ammonia (NH_3_) are in equilibrium, with the majority in the form of ammonium ion at physiological pH. In practice, the clinical laboratory measures the total concentration of NH_3_/NH_4_^+^, which is clinically referred to as “ammonia” or “blood ammonia.” In this paper, “ammonia” is used to represent the total concentration of the two forms, recognizing that there is not a clinical method to distinguish the precise proportion in any specific tissue nor sample.

Malnutrition leads to increased ammonia production via catabolism and resulting endogenous protein breakdown [9, 10]. Malnourished patients are at risk for developing secondary urea cycle dysfunction due to nutritional deficiencies (e.g., zinc and carnitine), resulting in decreased ammonia clearance [2, 11]. While malnutrition has been previously reported as a potential cause of hyperammonemia, it remains a diagnosis of exclusion. As a comprehensive work‐up is underway, patients are often offered a variety of treatments in an attempt to lower the ammonia level [10]. Restriction of protein intake is often the first step taken when another etiology is not identified [3]. Rifaximin and/or lactulose may address hyperammonemia by reducing ammonia‐producing bacteria and/or promoting ammonia excretion, respectively [6]. N‐carbamoyl glutamate and ammonia scavengers, such as sodium phenylbutyrate and sodium benzoate, are classic treatments for urea cycle defects that may also be utilized in hyperammonemia of unclear etiology [6]. Arginine or citrulline can also be used to promote nitrogen excretion via the urea cycle [12]. There is a growing body of evidence to show that an important aspect of treatment of hyperammonemia secondary to malnutrition is to induce anabolism with dextrose, lipids, and early reintroduction of protein [2, 10, 13, 14, 15].

This series presents cases of hyperammonemia in medically complex patients for whom common etiologies of hyperammonemia were excluded after extensive evaluations. All of these patients displayed clinical signs of malnutrition. We highlight the unique biochemical features of malnutrition‐related hyperammonemia, discuss possible mechanisms to explain the pattern, and propose a framework for managing these cases. We highlight hypoaminoacidemia and the appearance of pyroglutamic acid as potential biomarkers in patients with poor nutrition, suggesting that earlier supplementation of protein, rather than restriction, may lead to faster resolution of hyperammonemia.

## 2 Methods

This research was deemed exempt under IRB protocol number 24‐1007 through the Colorado Multiple Institutional Review Board. A retrospective chart review of patients who presented with hyperammonemia between July 2018 and July 2023 was conducted. Software within the electronic medical record was utilized to filter the records. There were 175 339 admissions during the designated time period; of those, 85 included the diagnosis code of hyperammonemia. This accounted for 62 unique patients. These 62 patients' charts were manually searched for an etiology of hyperammonemia. Based on a review of recent literature and known causes of hyperammonemia, the following findings resulted in exclusion from the case series: underlying inborn error of metabolism (citrullinemia, ornithine transcarbamylase deficiency, isovaleric acidemia, methylmalonic acidemia, hyperinsulinism hyperammonemia), valproic acid toxicity, portosystemic shunt, fibrolamellar hepatocellular carcinoma, hepatic fibrosis, liver dysfunction, history of a bone marrow transplant, biliary atresia, transient hyperammonemia of the newborn, and the presence of urease‐producing bacteria.

A total of three patients remained in the series. From the charts of these patients, various objective measures were collected: ammonia levels throughout their admission(s); patient weight; plasma amino acid profiles; urine organic acid profiles; plasma carnitine profiles; and zinc, phosphorous, and albumin values. Many of the laboratory values listed above were followed serially in conjunction with the ammonia‐lowering agents.

Urine organic acids and plasma amino acids were reviewed, looking for trends as various treatments were introduced. Urine organic acids were analyzed using gas chromatography mass spectrometry as described previously [16]. The only alterations made were: (1) the internal standard used was dimethylmalonic acid, and (2) chromatography was completed on a 30‐m column of DB‐17 (J & W Scientific, Folsom, CA). Qualitative urine organic acid chromatograms were individually reviewed for each patient, and the pyroglutamic acid level was noted relative to the internal standard. Pyroglutamic acid was identified as two peaks. Peak 1 (diTMS) was detected using ions 147, 156, 230, and 258 at 9.486 min while peak 2 (monoTMS) was detected using the ions 84, 157, and 186 at 10.1 min. To more objectively compare the concentration of pyroglutamic acid, the total ion chromatogram areas of peak 1 and peak 2 were summed and the total peak area was compared to dimethylmalonic acid at a known concentration. The final value was corrected for creatinine.

## 3 Results

### 3.1 Patient 1

A 5‐year‐old male with a history of traumatic brain injury, spastic cerebral palsy, epilepsy, and gastrostomy tube (G‐tube) dependence presented with somnolence. He was found to have hyperammonemia to 176 μmol/L (normal 21–50 μmol/L). ALT was mildly elevated (52 U/L; normal 10–41 U/L), but AST, PTT, and PT/INR were normal. His albumin was low (2.0 g/dL; normal 3.5–5.2 g/dL). The patient was noted to have hair pigment changes from a dark to a copper‐like appearance. One month prior, he was hospitalized with acute respiratory failure due to complex pneumonia complicated by vasopressor‐responsive septic shock in the setting of pseudomonas bacteremia. During that admission, he received multiple broad‐spectrum antibiotics.

**FIGURE 1:**
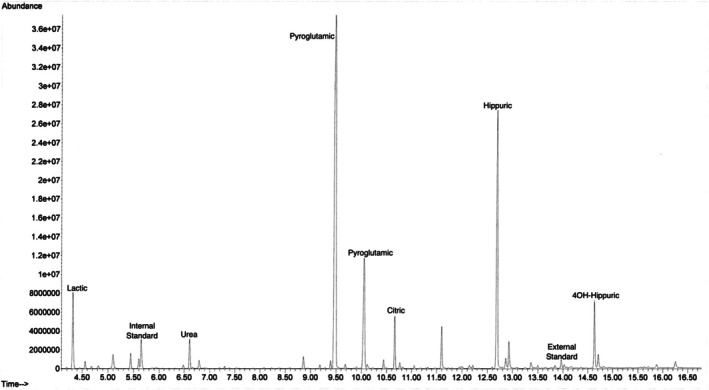
Chromatogram of urine organic acids obtained on admission for Patient 1. Note the two large pyroglutamic peaks.

The metabolism team was consulted due to hyperammonemia of unclear etiology. Intravenous sodium phenylacetate/sodium benzoate, arginine, and lactulose were initiated, and protein sources were withheld without improvement in hyperammonemia. Biochemical work‐up was performed to screen for inborn errors of metabolism. Urine orotic acid was normal. Urine organic acids showed two large peaks of pyroglutamic acid (when compared to dimethylmalonic acid at a known concentration, it showed two peaks: 21 047.5 and 5675.3 mmol pyroglutamic acid/mol creatinine (Figure 1)) and significant lactic aciduria. The patient had normal urine organic acids 3 years earlier. Plasma amino acids demonstrated many deficiencies (Table 1). Rapid whole genome sequencing did not show a cause of the hyperammonemia, and the remainder of the biochemical workup was not consistent with an inborn error of metabolism.

**TABLE 1:** Laboratory values of each patient including ammonia, amino acids, urine organic acids, and other nutrition labs.

		Patient 1	Patient 2	Patient 3—Admission 1	Patient 3—Admission 2
Ammonia (μmol/L)	At presentation	**176**	**112**	**331**	**162**
	Maximum	**416**	**187**	**331**	**176**
Amino acids (normal range in nmol/mL unless otherwise stated)	Date	1 day after presentation	7 days after presentation	Day of presentation	3 days after presentation
	Alanine (152–547)	**996**	191 (ref: 177–583)	317	175
	Arginine (10–140)	18	20 (ref: 15–128)	32	28
	Citrulline (1–46)	12	**7 (ref: 12–55)**	23	4
	Cystine (5–45)	12	5 (ref: 0–3)	**2**	**< 2**
	Glutamine (254–823)	**1257**	**831 (ref: 205–756)**	**1716**	302
	Glycine (127–341)	145	151 (ref: 151–390)	**348**	**88**
	Histidine (41–125)	95	**56 (ref: 72–124)**	115	55
	Isoleucine (22–107)	**9**	**4 (ref: 30–108)**	28	**5**
	Leucine (49–216)	**30**	**12 (ref: 72–201)**	**39**	**13**
	Lysine (48–284)	261	**50 (ref: 100–250)**	194	**41**
	Methionine (7–47)	**2**	**3 (ref: 10–42)**	**4**	**5**
	Ornithine (10–163)	64	**37 (ref: 48–195)**	96	34
	Phenylalanine (26–91)	**9**	**8 (ref: 35–85)**	**18**	34
	Proline (59–369)	244	274 (ref: 97–329)	**459**	188
	Serine (69–187)	**47**	**35 (ref: 58–181)**	139	**22**
	Taurine (10–170)	26	**48 (ref: 54–210)**	76	32
	Threonine (35–226)	81	**29 (ref: 54–210)**	90	**13**
	Tryptophan (5–75)	**< 2**	**< 2 (ref: 30–95)**	**< 2**	**< s2**
	Tyrosine (24–115)	**3**	**4 (ref: 34–112)**	**15**	**6**
	Valine (74–321)	87	**22 (ref: 119–336)**	77	**33**
Urine organic acids	Date	1 day after admission	7 days after admission	Day of admission	8 days after admission
	Pyroglutamic acid peak	2 large peaks	1 large peak	1 large peak	2 large peaks
	Pyroglutamic peak quantitation (mmol pyroglutamic acid/mol creatinine)	Peak 1: 21047.5, Peak 2: 5675.3	Peak 1: 3835.5, Peak 2: 725.2	Peak 1: 170.1, Peak 2: 19.6	Peak 1: 57855.7, Peak 2: 9642.6
Other nutrition labs	Zinc level (μg/dL, normal 60–120)	**40**	71	**51**	n/a
	Albumin g/dL	**2.0 (ref: 3.5–5.2)**	**2.2–2.6 (ref: 3.5–5.0)**	**1.5–1.7 (ref: 3.7–5.6)**	**1.7–2.4 (ref: 3.5–5.2)**

*Note:* Values that are grey‐scaled and bolded are values below the lower limit of normal. Values that are bolded only are values above the upper limit of normal. Note that there are unique reference ranges for Patient 2 based on differences in age.

Abbreviation: ref, reference.

Of note, the patient had a 12% weight loss in the month leading up to his admission. His Z‐score for weight around the time of admission was −2.3, and 7 months prior, his Z‐score was −1.7. Due to concern for severe protein malnutrition, total parenteral nutrition (TPN) was initiated with 1.7 g/kg/day protein, 1 g/kg/day intralipids, and a glucose infusion rate of 6 mg/kg/min. The ammonia level normalized soon after TPN was initiated. The patient was transitioned to G‐tube feeds, enteral ammonia scavengers (sodium benzoate and sodium phenylbutyrate), and arginine. He was also treated with a course of rifaximin to treat possible small intestinal bacterial overgrowth. Repeat plasma amino acids demonstrated persistent deficiencies, so enteral protein was increased to 2.0 g/kg/day. The patient's ammonia level remained normal, so the ammonia‐lowering agents were discontinued in the following order: sodium phenylbutyrate, arginine, rifaximin, and sodium benzoate. Hyperammonemia did not recur.

### 3.2 Patient 2

A 22‐year‐old female with a known, progressive genetic neurodevelopmental disorder, restrictive lung disease, tracheostomy and ventilatory dependence, G‐tube dependence, spastic cerebral palsy, scoliosis status‐post posterior spinal fusion, and epilepsy, requiring multiple anti‐epileptic medications (not including valproic acid) and vagal nerve stimulator presented with lethargy and fluid‐refractory septic shock. On laboratory evaluation, she was found to have hyperammonemia to 112 μmol/L. AST was mildly elevated at 58 U/L (normal 10–41 U/L), but ALT, PTT, PT/INR were normal. Albumin was low (1.0–2.0 g/dL; normal 3.5–5.2 g/dL). She was noted to have a body mass index of 19.48 kg/m^2^, hair pigment change from brown to blonde, and a 9‐month history of alopecia. Six months prior, the patient was admitted for lethargy, bradycardia, and hypothermia. She was treated with broad‐spectrum antibiotics before being diagnosed with MSSA tracheitis.

**FIGURE 2:**
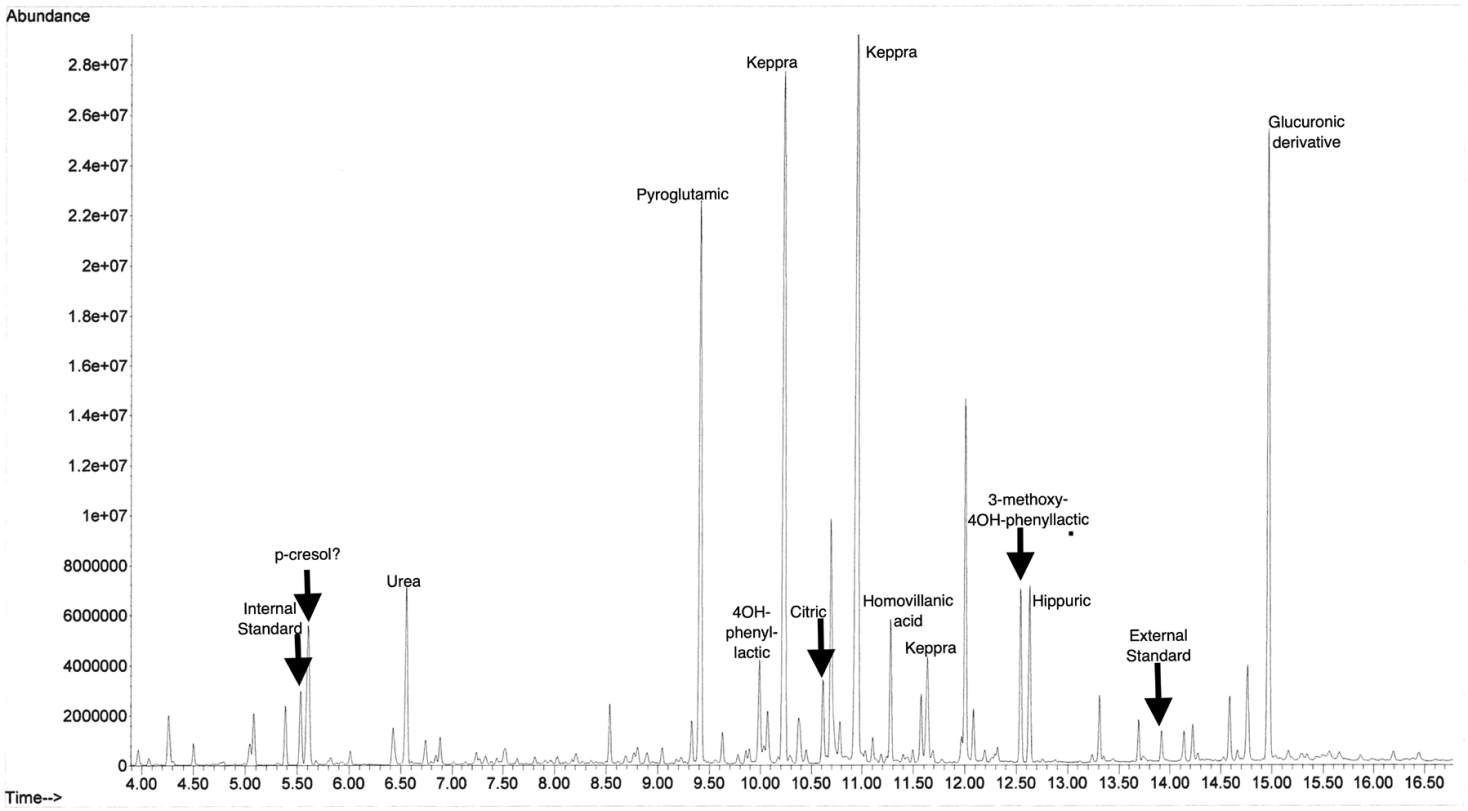
Chromatogram of urine organic acids obtained on admission for Patient 2. Note the large pyroglutamic peak.

The patient was admitted to the hospital for management of septic shock, and she was started on vancomycin and cefepime given mixed flora in urine and tracheostomy cultures. The metabolism team was consulted due to hyperammonemia of unclear etiology. Intravenous sodium phenylacetate/sodium benzoate, rifaximin, and lactulose were initiated, and protein sources were withheld. Urine orotic acid was normal, and urine organic acids showed one large peak of pyroglutamic acid (when compared to dimethylmalonic acid at a known concentration, it showed two peaks: 3835.5 and 725.2 mmol pyroglutamic acid/mol creatinine (Figure 2)). Plasma amino acids demonstrated gross deficiencies, with very low essential amino acids and citrulline (Table 1). Rapid whole genome sequencing did not identify any new pathogenic variants to explain her hyperammonemia, and her biochemical workup did not indicate an inborn error of metabolism.

The patient's ammonia level remained in the 100's despite 24 h of intravenous sodium phenylacetate/sodium benzoate. Thus, it was discontinued, and N‐carbamoyl glutamate, rifaximin, and intravenous arginine were initiated. Protein was gradually reintroduced through her G‐tube feeds at 1.3 g/kg/day. N‐carbamoyl glutamate did not result in ammonia reduction, so a trial of enteral sodium benzoate and sodium phenylbutyrate was initiated, along with continuation of intravenous arginine and rifaximin. Lactulose was inconsistently administered due to intermittent diarrhea. By Day 10 of hospitalization, the patient's ammonia normalized. She was discharged on sodium benzoate, sodium phenylbutyrate, arginine, and rifaximin. These were all eventually discontinued.

In retrospect, it was noted that the patient had lost 16 kg over the 2 years preceding her hyperammonemia admission. Repeat plasma amino acids normalized upon follow‐up 9 months after discharge with an improving BMI. Hyperammonemia did not recur.

### 3.3 Patient 3

A 6‐year, 4‐month‐old male with a severe form of epidermolysis bullosa and G‐tube dependence presented with altered mental status, abnormal movements, and lip smacking. He was found to have hyperammonemia to 331 μmol/L. AST/ALT, PTT, and PT/INR were normal. Albumin was low (1.5–1.7 g/dL; normal 3.5–5.2 g/dL). He was SARS‐CoV‐2 PCR positive. MRI of the brain was normal outside of minimal cerebral volume loss secondary to a remote hypoxic ischemic injury.

**FIGURE 3:**
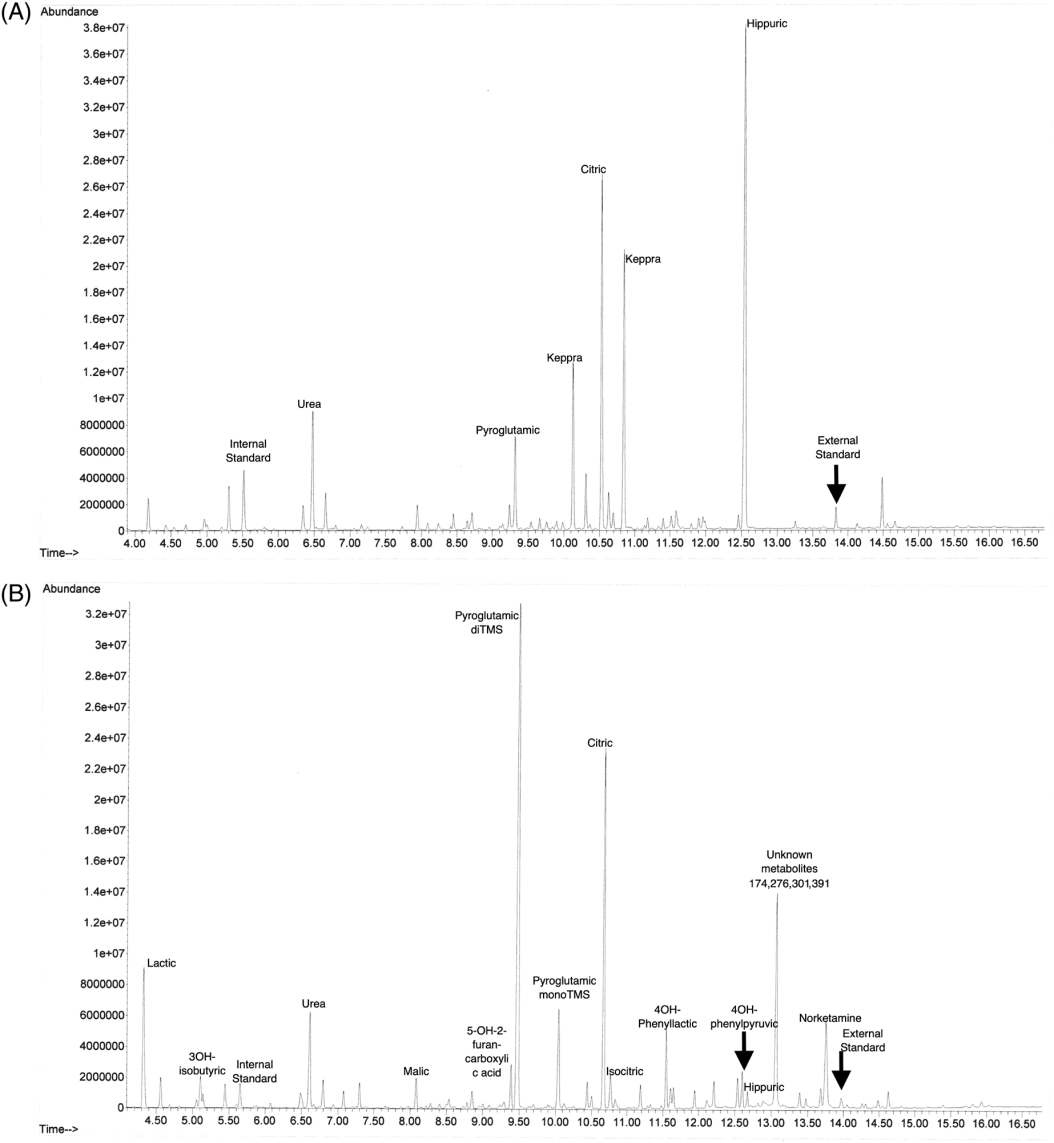
Chromatogram of urine organic acids obtained on the first (A) and second (B) admissions for Patient 3. Note the large pyroglutamic peak in his first admission and the two large peaks in his second admission.

The metabolism team was consulted due to hyperammonemia (331 μmol/L, normal 21–50) of unclear etiology, and intravenous sodium phenylacetate/sodium benzoate were initiated. Protein sources were withheld, and the patient was placed on dextrose‐containing intravenous fluids. Over the next 48 h, the patient's ammonia decreased; however, he then developed culture‐negative fluid‐refractory shock and an increasing ammonia to 298 μmol/L. Continuous renal replacement therapy (CRRT) and N‐carbamoyl glutamate were initiated, as well as rifaximin for possible small intestine bacterial overgrowth. Urine orotic acid was normal, and urine organic acids demonstrated one large peak of pyroglutamic acid (when compared to dimethylmalonic acid at a known concentration, it showed two peaks: 170.1 and 19.6 mmol pyroglutamic acid/mol creatinine (Figure 3A)). Plasma amino acids showed significant deficiencies (Table 1). While the patient was on CRRT, his ammonia level was resistant to clearance. Abdominal ultrasound with doppler and CT venogram were normal, ruling out portosystemic shunt. Rapid whole genome sequencing did not identify any new pathogenic variants to explain his hyperammonemia, and biochemical work‐up did not indicate an inborn error of metabolism.

Protein‐containing G‐tube feeds were started around 1 week into his admission, and the patient's ammonia normalized slowly. He was discharged home after a three‐week admission on a diet that consisted of 126 kcal/kg/day and 4.4 g/kg/day of protein. In the setting of feeding intolerance and multiple hospitalizations, his weight fluctuated significantly during this period: 19.3 kg at 6 years, 4 months; 14.2 kg at 6 years, 6 months; and 17.5 kg at 6 years, 7 months. His Z‐score for weight during this admission was −1.7.

Seven months prior to this first admission, the patient was admitted for encephalopathy, vasopressor‐refractory septic shock, significant hypoglycemia leading to seizures, and hypotensive ischemic stroke requiring broad‐spectrum antibiotics. His hospital course was prolonged, and he was discharged after 1 month, having lost 0.7 kg. An ammonia level was not drawn. Given his underlying EB diagnosis, the patient was discharged on a caloric‐ and protein‐dense diet, similar to his baseline, which provided 123 kcal/kg/day total with 3.8 g/kg/day of protein. Leading up to his first admission, he experienced frequent feeding intolerance.

Unfortunately, the patient presented to the hospital twice more in the subsequent months with altered mental status, nausea, emesis, constipation, and hyperammonemia. Each time, his ammonia initially improved with intravenous dextrose‐containing fluids, protein restriction, lactulose, and N‐carbamoyl glutamate, yet his ammonia would gradually increase again. Urine organic acids persistently demonstrated two large peaks of pyroglutamic acid (when compared to dimethylmalonic acid at a known concentration, it showed two peaks: 57855.7 and 9642.6 mmol pyroglutamic acid/mol creatinine (Figure 3B)). Plasma amino acids continued to show hypoaminoacidemia (Table 1). In each admission, treatment with glycerol phenylbutyrate and repeated courses of rifaximin were attempted without success. Notably, during later hospitalizations, protein was reintroduced and liberalized much sooner, and his admissions were shorter. The patient slowly gained weight, and after his third admission for hyperammonemia, his G‐tube regimen provided 100 kcal/kg/day and 2 g/kg/day of protein; this was accompanied by improvement in his feeding tolerance. Follow up plasma amino acids normalized. Hyperammonemia did not recur.

## 4 Discussion

We present a case series of hyperammonemia due to malnutrition where the simultaneous presence of hyperammonemia, hypoaminoacidemia, and pyroglutamic aciduria were noted. Clinical similarities among these patients included physical evidence of malnutrition and/or nutritional deficiencies (e.g., acute/chronic weight loss, hair pigment changes), G‐tube use, and an admission for infection within the prior year requiring broad‐spectrum antibiotics. In all three cases described, multiple treatments were attempted, but the most effective treatment and preventative measure of hyperammonemia was initiation of adequate nutrition—specifically protein—to promote anabolism.

Several laboratory findings suggested malnutrition. All patients had low albumin. This may be the first result available, so it is important for clinicians to note this as a clue for malnutrition‐related hyperammonemia. Zinc levels were low in Patients 1 and 3 and on the low end of the normal range in Patient 2 (Table 1). Hypoaminoacidemia was present as evidenced by at least 7 amino acid deficiencies, namely tyrosine, tryptophan, methionine, and branched‐chain amino acids (Table 1).

Particular deficiencies in plasma amino acids are characteristic of malnutrition and have been previously reported in patients with noncirrhotic hyperammonemia [2]. The most common deficiencies occur in the branched‐chain amino acids; low methionine has also been reported in patients with hyperammonemia with carnitine deficiency (not consistently seen in the patients presented) [2, 17]. In the same case series of patients with noncirrhotic hyperammonemia, all of the patients with both a diagnosis of unexplained, acquired urea cycle disorders and a zinc level drawn (*n* = 6) had zinc deficiency [2]. In both human and animal studies, zinc deficiency correlates with hyperammonemia, and replacement of zinc corrects the hyperammonemia [11, 18, 19, 20]. Ornithine transcarbamylase protein homotrimers bind zinc; however, the specific impact of zinc deficiency on enzyme activity in vivo is not clear and could potentially be related to a role in bridging or clustering of the proximal urea cycle enzymes (N‐acetylglutamate synthase, carbamoyl phosphate synthetase I, and ornithine transcarbamylase) on the mitochondrial inner membrane [21].

We found that pyroglutamic acid was elevated in this series of individuals with malnutrition‐related hyperammonemia. Previous studies have shown that pyroglutamic acid (also referred to as 5‐oxoproline) may be detected in the urine in patients with hyperammonemia due to urea cycle defects, apparently related to the consumption of glutathione during metabolic crises [22, 23]. The presence of pyroglutamic acid has also been associated with patients on artificial diets (e.g., formula via G‐tube), with malnutrition, and with the use of acetaminophen and antibiotics such as flucloxacillin and netilmicin [24, 25, 26]. These agents inhibit enzymes within the gamma‐glutamyl cycle, whose rate limiting step is 5‐oxoprolinase, which transforms 5‐oxoproline to glutamate [27, 28, 29, 30]. In the three patients presented here, there was no evidence to suggest that their diets nor use of specific medications were the cause of increased pyroglutamic acid levels. A recent review suggests a variety of other associations between an elevated pyroglutamic acid such as vigabatrin, cancer, and chronic pain [26]. In this same review, low albumin and hyperammonemia were noted [26]. We suggest that while malnutrition was a primary driver of the hyperammonemia, it is likely that their hyperammonemia was multifactorial in nature.

**FIGURE 4:**
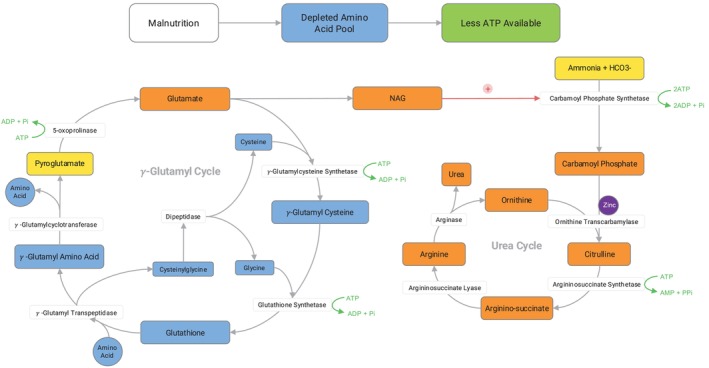
Authors' hypotheses as to why the pattern of hypoaminoacidemia, hyperammonemia, and pyroglutamic acid were observed in patients with malnutrition. In the gamma‐glutamyl cycle, specifically low methionine results in low cysteine, an amino acid critical for glutathione synthesis. Many amino acids are necessary for transamination reactions, creating glutamate, which is used to create N‐acetylglutamate, which upregulates carbamoyl synthetase I. Without sufficient glutamate, there is reduced function of both the urea cycle and gamma‐glutamyl cycle, resulting in elevated ammonia and pyroglutamic acid, respectively.

Hypoaminoacidemia may underlie the development of hyperammonemia in malnutrition due to an overall energy‐deplete state that ultimately results in less ATP available for critical pathways such as the transamination reactions, gamma‐glutamyl cycle, and the urea cycle (Figure 4). The absence of methionine, which is a precursor to cysteine production, a critical amino acid for glutathione synthesis within the gamma‐glutamyl cycle, may contribute to the elevation of pyroglutamic acid [26, 31]. Amino acids are necessary for transamination reactions, which ultimately produce glutamate, which in turn is used to synthesize NAG [29]. NAG upregulates carbamoyl phosphate synthetase I, one of the first enzymes within the urea cycle [29]. The deficiency of glutamate has been proposed as a mechanism of decreased urea cycle function and therefore increased risk of hyperammonemia in patients with malnutrition [10, 22, 32]. However, more research is needed to refine or replace these hypotheses. Additionally, this study is limited by the number of patients included, so further studies are needed to validate the simultaneous appearance of hyperammonemia, hypoaminoacidemia, and pyroglutamic acid in the setting of malnutrition. Another limitation is the lack of follow‐up urine organic acids to confirm the resolution of pyroglutamic aciduria once the nutrition had been optimized.

If malnutrition is thought to be a primary driver of hyperammonemia, the reintroduction of protein is paramount in the approach to management [2]. Early reintroduction of protein in the setting of hyperammonemia is antithetical to the classic teaching of hyperammonemia management, often waiting as long as 48 h if ammonia remains elevated. However, in this case series, protein reintroduction was the only intervention that resulted in sustained normalization of ammonia levels. Of course, performing molecular sequencing and a broad workup simultaneously to exclude other causes of hyperammonemia (e.g., portosystemic shunt, urease‐producing bacteria) is necessary to guide treatment in each patient.

## 5 Conclusion

The combination of (1) evidence of protein insufficiency (low serum albumin, low amino acids, and pyroglutamic aciduria); (2) lack of plasma amino acid abnormalities indicating primary urea cycle enzyme defects (elevated/deficient citrulline, presence of argininosuccinic acid, or elevated/deficient arginine); and (3) urine organic acids without significant orotic acid/uridine elevations nor evidence of organic acidemias could allow us to be clinically confident in the decision to deliver protein early in the treatment period, leading to more rapid resolution of hyperammonemia in cases of malnutrition. Close monitoring of ammonia while doing this reassures against inadvertently worsening the hyperammonemia. This case series suggests that early reintroduction of protein can be safely and appropriately delivered with confidence after measuring plasma amino acids and urine organic acids.

## Author Contributions

**M. M. Crenshaw:** conceptualization, data curation, manuscript writing, manuscript editing, formal analysis, investigation. **O. M. D'Annibale:** data curation, manuscript writing, manuscript editing, formal analysis, investigation, methodology. **V. Martucci:** data curation, formal analysis, investigation. **S. Gracie:** data curation, manuscript editing, formal analysis, investigation. **A. Kochhar:** data curation, manuscript editing, formal analysis, supervision. **J. Stansauk:** data curation, formal analysis. **A. Larson:** conceptualization, data curation, investigation, supervision. **P. Baker II:** conceptualization, manuscript editing, data curation, supervision. **C. Peck:** data curation, manuscript writing, manuscript editing, formal analysis, investigation, methodology. **T. Wood:** conceptualization, data curation, manuscript writing, manuscript editing, formal analysis, investigation, methodology, supervision. **A. El‐Gharbawy:** manuscript editing, formal analysis, investigation, methodology, supervision. **S. McCandless:** conceptualization, data curation, manuscript writing, manuscript editing, formal analysis, investigation, supervision. **M. K. LoPiccolo:** conceptualization, data curation, manuscript writing, manuscript editing, formal analysis, investigation, methodology, supervision.

## Funding

The authors have nothing to report.

## Conflicts of Interest

The authors declare no conflicts of interest.

## Data Availability

The data that support the findings of this study are available on request from the corresponding author. The data are not publicly available due to privacy or ethical restrictions.
